# Effects of Surgery on the 30-Day Survival Rate in Spontaneous Supratentorial Intracerebral Hemorrhage

**DOI:** 10.3390/brainsci11010005

**Published:** 2020-12-23

**Authors:** Adrian Balasa, Dana Ghiga, Razvan-Sebastian Andone, Ancuta Elena Zahan, Ioan Alexandru Florian, Rares Chinezu

**Affiliations:** 1 Department of Neurosurgery, George Emil Palade University of Medicine, Pharmacy, Science and Technology, 540142 Tîrgu Mureș, Romania; rares.chinezu@umfst.ro; 2 Department of Neurosurgery, Tîrgu Mureș Clinical Emergency County Hospital, 540136 Tîrgu Mureș, Romania; 3 Department of Medical Informatics and Biostatistics, George Emil Palade University of Medicine, Pharmacy, Sciences and Technology, 540142 Tîrgu Mureș, Romania; dana.ghiga@umfst.ro; 4 Department of Neurology, Tîrgu Mureș Clinical Emergency County Hospital, 540136 Tîrgu Mureș, Romania; sebastian.andone@umfst.ro; 5 Department of Histology, George Emil Palade University of Medicine, Pharmacy, Science and Technology, 540142 Tîrgu Mureș, Romania; ancutza_ct@yahoo.com; 6 Department of Neurosurgery, Iuliu Haţieganu University of Medicine and Pharmacy, 400012 Cluj-Napoca, Romania; florian.ioan.alexandru@gmail.com; 7 Department of Neurosurgery, Cluj-Napoca Clinical Emergency County Hospital, 400006 Cluj-Napoca, Romania

**Keywords:** stroke, hypertensive intracerebral hemorrhage, intracerebral hemorrhage, neurosurgical intervention, ICH score

## Abstract

*Background:* Spontaneous intracerebral hemorrhage (ICH) is a severe form of stroke. The efficacy of surgery as ICH treatment is controversial. We sought to compare the 30-day postoperative mortality rate between patients with surgically and medically treated ICH; Methods: This prospective study enrolled patients consecutively diagnosed with ICH and treated between 2017 and 2019. Patients meeting the study surgical indications were assigned to either surgical or medical treatment. The relationship between Glasgow Coma Scale (GCS) score, age, ICH location, ICH volume, and 30-day mortality was analyzed. Results: A total of 174 ICH patients were enrolled in this study. Of these, 136 met the surgery criteria; 65 of these underwent surgery (Group A), and 71 received medical treatment (Group B). Age and ICH location did not modify mortality. Although surgery did not overall improve mortality some better postsurgical outcomes were observed among patients surgically treated with GCS scores of at least 10 points and ICH volumes between 30 to 50 mL; *Conclusions:* Despite achieving an immediate reduction in intracranial pressure, surgery seems to be advantageous only for patients with ICH volumes between 30 to 50 mL and GCS scores of 10 points or higher;

## 1. Introduction

Spontaneous, nontraumatic intracerebral hemorrhage (ICH) is the most common and severe type of hemorrhagic stroke and is associated with significant mortality and morbidity rates worldwide [1,2,3,4,5]. ICH diagnosis is commonly made by conducting emergency computed tomography (CT) imaging and should always exclude secondary causes of hemorrhage, such as vascular or tumoral pathology [6,7,8]. Occult causes of secondary hypertension should also be considered, especially in young patients [9].

The surgical removal of a hematoma, in theory, should confer benefits due to the lower resultant intracranial pressure (ICP) and the reduced occurrence of secondary lesions associated with hematoma degradation [10]. However, the role of surgery in ICH treatment remains controversial as two major, international, randomized studies (STICH I and STICH II) were unable to identify any apparent benefits of surgery over the medical treatment in ICH [11,12,13]. Recently, these studies, due to their patient selection structure, have been challenged [14,15], deciding to undertake surgery being a continued matter of debate [16].

### Aims and Scope

In this study, we aimed to compare the 30-day mortality rate between patients diagnosed and treated for supratentorial ICH using either surgical or medical means.

## 2. Materials and Methods

### 2.1. Patients

This prospective study was performed between 2017 and 2019 and enrolled all patients admitted with supratentorial ICH who were medically or surgically treated in our stroke unit at the Tîrgu Mureș Emergency Clinical County Hospital, Romania. Approval for this study was obtained from the Institutional Ethics Committee of the Tîrgu Mureș University of Medicine and Pharmacy (ethic approval code: 182/2016). Consent for study participation was given by each patient’s next of kin.

### 2.2. Study Inclusion

Patients were included if presenting with spontaneous supratentorial ICH and if consent for their participation in the present study was given by their next of kin. Patients receiving anticoagulant treatment, with known hemopathies or with other medical conditions that could affect coagulation (e.g., hemophilia, platelet count < 80,000), and those having significant intraventricular hemorrhage (more than minimal blood in ventricles) were excluded from this study. Because we consider it a different pathology, secondary hemorrhage due to vascular or tumoral pathologies was excluded.

#### 2.2.1. Surgical Indication

The study participants considered eligible for surgical intervention were then divided into two groups according to the treatment received—surgical or medical—which was decided by the next of kin.

As no clear guidelines could be identified in the literature regarding surgical intervention indications [5], we proposed our own. Inclusion criteria, such as ICH volume and GCS score, were modeled following the multicenter metanalysis results of Gregson et al. [17]

Surgery was indicated when patients met all of the following criteria: (1) hematoma volume between 30 and 70 mL; (2) a mass effect of the hematoma and surrounding edema greater than 5 mm on midline structures; and (3) a neurological status degradation of at least two points on the Glasgow Coma Scale (GCS), with a total score of 7 to 13 points.

#### 2.2.2. Choice of Surgical or Medical Treatment

**Figure 1:**
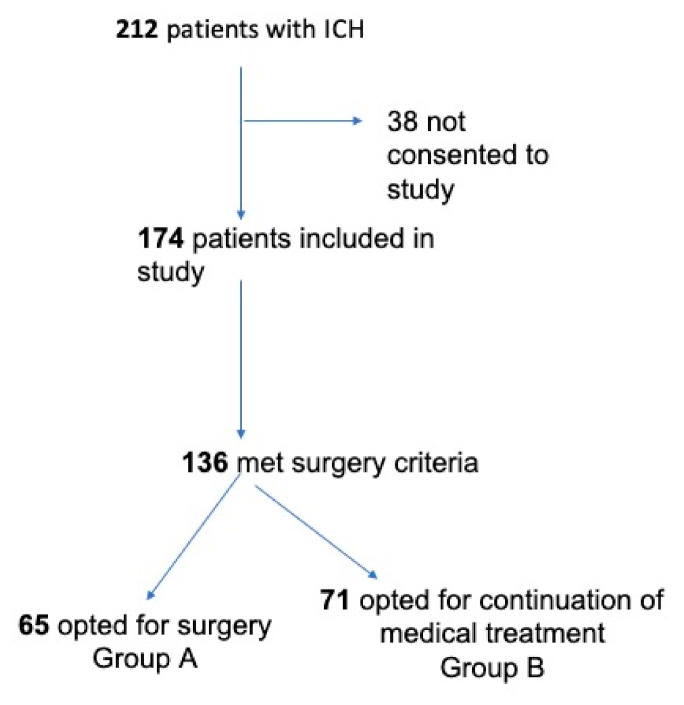
Inclusion criteria and the final study groups.

Following decision-making regarding whether a patient was eligible for surgery, either surgical treatment (Group A) or medical treatment (Group B) was applied, following the consent given by the next of kin, who actively chose one of the study treatments arms (Figure 1).

Counseling was offered to the next of kin by a joint team of neurologists and neurosurgeons, who provided complete information regarding the current state of knowledge of ICH’s medical and surgical treatment and presented the presumed advantages and disadvantages of each treatment to support informed decision-making. Individuals who were next of kin for the selected patients were informed that not opting for a surgical intervention did not qualify as a withdrawal of better treatment. They were also informed of their legal right to refuse surgery or to reconsider surgery if they initially chose medical treatment, which would result in the subsequent removal of the patient from the study (Figure 1).

Those who did not consent to participate in this study received similar medical or surgical treatment. In these cases, if surgical treatment was considered, indications and surgery were performed by senior neurosurgeons who were not involved in the present study.

### 2.3. Neuroimaging Evaluation

All patients were scanned using a multichannel/multidetector CT scanner (Siemens Somatom, Siemens Healthineers AG, 80333 Erlangen, Germany) to obtain images with 0.5-mm slice thickness. The hematoma volume was estimated using the ellipsoidal method [4/3π (a.b.c)], as described by Broderick et al. [18]. Cases with suspicion of hemorrhage secondary to intracerebral vascular or tumor pathologies were differentially diagnosed using angio-CT or contrast-enhanced CT.

### 2.4. Scoring

ICH was considered superficial when present at any lobar location, whereas hemorrhage in the basal ganglia was considered deep ICH [4].

Patients were graded as having medium-sized ICH when ICH volume was in between 30–49.99 mL and large ICH for ICH volumes of 50 to 70 mL. Neurological status was considered moderately affected for GCS 10 to 13 points, while severely affected was considered for patients with GCS of 7 to 9 points.

### 2.5. Medical Management

The medical treatment was provided by the Neurology Department of the Tîrgu Mureș Emergency Clinical County Hospital, an established tertiary center for stroke pathology. Patients were medically treated for ICH following the “Guidelines for the Management of Spontaneous Intracerebral Hemorrhage” from the American Heart Association/American Stroke Association [5] and “European Stroke Organization guidelines for the management of spontaneous intracerebral hemorrhage” [19]

The department has all the necessary resources to treat patients with ICH (consultant neurologists specialized in stroke pathology, nurses with a qualification in this field, and a multidisciplinary rehabilitation team, including speech and dysphagia specialist).

Regarding the medical treatment and management, the following were applied: continuous neurological assessment of consciousness, continuous cardiopulmonary monitoring, in cases requiring such continuous airway maintenance was performed, elastic stockings were used to prevent thromboembolism, prolonged bed rest complications were prevented through positioning and mobilization.

For acute ICH management, an early intensive blood pressure-lowering treatment was given in patients presenting with systolic blood pressure >220 mmHg by continuous intravenous infusion with nicardipine. Patients with lower values of blood pressure received oral treatment until the systolic blood pressure reached 140 mmHg.

Patients with perihematomal edema received hyperosmotic therapy with mannitol 20%, 250 mL 1.25 g/kg over 30–60 min, with strict surveillance of renal seric values.

Antiepileptic drugs were administered in patients having seizures. Levels of seric glycemia were actively measured and kept within the normal range.

No recombinant FVIIa was used in our patients as no clinical benefits are known in ICH patients without low levels of FVII.

Daily fluid balance was calculated in ICH patients and kept between −0.5/+1 L. Patients with secondary ICH to anticoagulant therapy were excluded from the study, and previous antiplatelet medication was discontinued for three weeks from ICH onset.

Medical complications were managed as follows. Antibiotics were administered when pneumonia, urinary infection, and sepsis were diagnosed. Low molecular heparin was administered in selected cases as prevention treatment or when pulmonary embolism and deep vein thrombosis were diagnosed. Nasogastric tubes were placed in patients with dysphagia and aspiration risk. Close cardiac surveillance and appropriate treatment were given in patients with heart failure, ventricular arrhythmias, and atrial fibrillation. Other medical complications, such as hyponatremia, gastrointestinal bleeding, or acute renal failure, were accordingly treated.

### 2.6. Surgical Management

**Figure 2:**
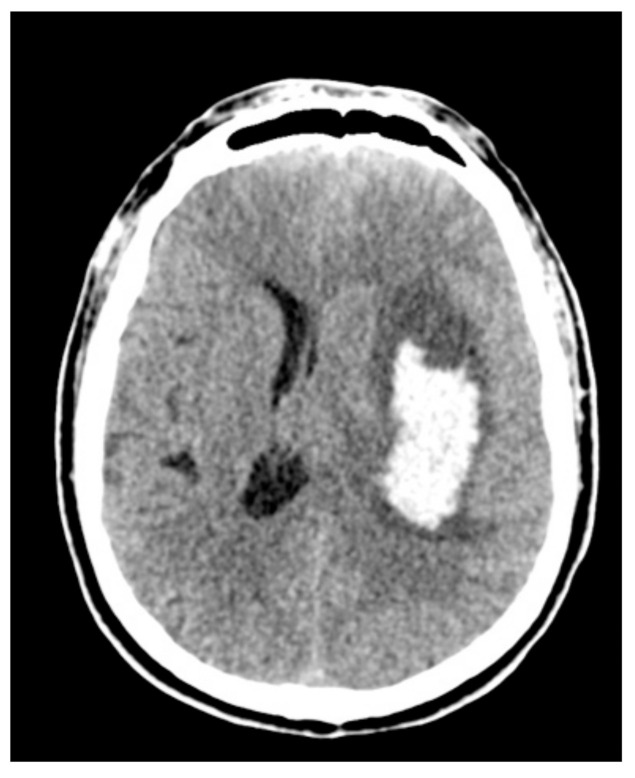
The preoperative aspect of a deep-seated intracerebral hemorrhage (ICH).

**Figure 3:**
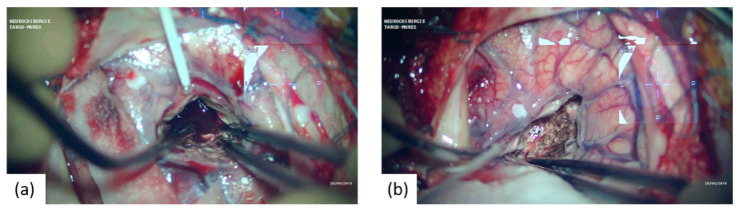
Microsurgical intraoperative aspects showing: (**a**) approach and initial aspect of the ICH; (**b**) after the ICH evacuation.

**Figure 4:**
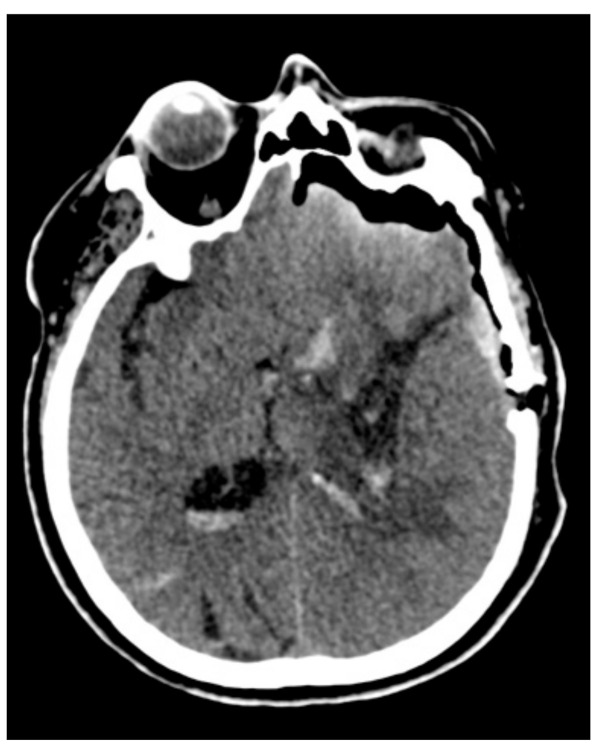
Control computed tomography (CT) after the microscopic minimally invasive subtotal evacuation of the clot.

All surgeries were performed under general anesthesia, and all patients received surgery via a minimal craniotomy with navigation guidance (Curve 2.1; Brainlab, 81829 Munich, Germany). An operative microscope was used during all surgeries (Zeiss S88; Carl Zeiss AG, 73447 Oberkochen, Germany or Leica OHX; Leica Microsystems, 35578 Wetzlar, Germany). Surgery was performed following an adapted “keyhole” technique, as initially described by Tsementzis [20], combined with a retractor-less “dynamic retraction” technique adapted from the work of Spetzler et al. [21]. The approach to the hematoma was performed under neuronavigation guidance and with image injection in the surgical microscope (Captiview; Leica Microsystems, 35578 Wetzlar, Germany). The approach trajectory was chosen to be as short as possible by using the trans-sulcal or trans-Sylvian approaches while, at the same time, avoiding eloquent areas. Significant attention was given to the dissection of sulci and fissures to minimize the surgical sacrifice of the cerebral parenchyma. In all cases, minimal corticectomy was sought and was achieved by combing the angulation of the microscope with the surgical table rotation. Complete hematoma reduction was not considered the surgical endpoint as we opted for a subtotal evacuation of the ICH clot volume, thus allowing for the reduction in ICP and diminishment of secondary cerebral injury, at the same time, minimizing brain manipulation and surgical time (Figure 2, Figure 3 and Figure 4).

### 2.7. Statistical Analysis

Statistical analysis was conducted using descriptive statistics (frequency, percentage, mean, median, and standard deviation) and inferential statistics elements. The Shapiro–Wilk test was applied to determine the distribution of the analyzed data series. For comparing averages between the two treatment arms, the Student’s *t-*test and the Mann–Whitney U test were applied. The chi-squared test was applied to determine associations between qualitative variables. The significance threshold was set at a *p*-value of 0.05. Statistical analysis was performed using GraphPad Prism 8 (GraphPad Software, San Diego, CA, 92108, USA) and IBM SPSS Statistics for Windows, version 27 (IBM Corp., Armonk, NY, 10504, USA).

## 3. Results

### 3.1. ICH Clinical Characteristics

Between 2017 and 2019, a total of 212 patients were admitted for spontaneous ICH. In 38 cases, consent to enroll the patient in this study was not given, resulting in 174 cases enrolled in our current ICH study group. Among these 174 enrolled cases, a total of 136 patients met the criteria for the surgical indication. Consent for surgery was obtained in 65 cases (Group A), whereas the remaining 71 opted for medical treatment (Group B) (Figure 1).

The mean age of the ICH study group was 63.47 (±13.76) years. No differences were observed between the mean ages (61.75 vs. 68.73 years) of Groups A and B.

The rates of associated comorbidities varied, with the most common being high blood pressure, which was identified in 72.30% (*n* = 47) of patients from Group A and 69.01% (*n* = 49) of Group B patients.

Other comorbidities were represented by cardiac diseases, diabetes, and obesity, but no significant statistical differences were seen between Groups A and B (Table 1).

The median GCS score for the surgical-indication group was 9 points. No statistically significant difference in the GCS score was observed between Groups A and B.

Regarding deep ICH location, this was identified in 34.55% (*n* = 47) of all cases with surgical indication. Deep ICH was identified in 30.76% of cases in Group A (*n* = 20) and 38.02% (*n* = 27) of cases in Group B. No significant differences were observed in this regard between any of the study groups (*p* = 0.373).

The mean ICH volume for the surgical indication group was 53.72 mL. Similar mean ICH volumes were calculated in Group A (56.62 mL) and Group B (50.82 mL).

### 3.2. Mortality of Surgically and Medically Treated Patients

There were no differences in mortality due to age or comorbidities between Groups A and B (*p* = 0.789).

In the whole surgical indication group, the 30-day mortality rate was 69.85% (n = 95), whereas that in Group A was 69.23 (*n* = 45) and that in Group B was 70.42% (*n* = 50) (*p* = 0.879752).

Mortality for deep-seated ICH was of 75% (*n* = 15) in Group A and 85.13% (*n* = 23) in Group B_._ There was no difference in mortality between deep and superficial surgically treated cases (*p* = 0.501). (Table 2)

There was no difference in mortality between moderate (30–50 mL) or large (50–70 mL) ICH volume between study groups (*p* = 0.413).

Severe neurological impairment (GCS 7–9) was associated with higher mortality when compared to moderate neurological impairment (GCS 10–13) (*p* = 0.0132)

When examining the association between neurological status, ICH volume, and mortality, we found no differences in mortality between the studied groups.

Post-hoc analysis of admission GCS and ICH volume revealed significantly lower mortality in the surgical group with GCS 10–13 and ICH volume 30–50 mL (*p* = 0.0294) (Table 3).

## 4. Discussion

ICH represents a critical component of stroke, with an incidence rate of up to 50% and a significantly high 30-day mortality rate of approximately 40% in developing countries, such as Romania [3,22]; however, no effective treatment has yet been identified [23].

As other authors observed [10,24], we consider that the 30-days mortality is a good indicator of the short-term, immediate, surgical, or medical care provided to ICH patients. Meanwhile, longer follow-up is dependent mostly on the quality of care and development of the health system. Unfortunately, not all our patients have access to similar rehabilitation facilities, significantly biasing our long-term results and limiting our study to the shorter follow up.

We opted to perform a prospective study. For those patients who qualified for surgical intervention, relatives were allowed to decide the course of action after being provided with objective information regarding both treatment options (surgical and medical).

Our surgical-indication was oriented according to the results of Gregson et al. [17], who reported, in their meta-analysis of surgically treated supratentorial ICH, better outcomes among patients with GCS scores of 9 points or higher and ICH volumes of 20 to 50 mL. Nevertheless, volumes of 20 to 30 mL rarely do give significant mass effect; as such, we chose to exclude them from the present study while at the same time opting to include larger volumes up to 70 mL to check if surgery has benefits for larger volumes.

Grading the severity of neurological deficit is challenging in ICH [25]. The original ICH score is still recommended as an adequate prognostic tool [19], despite being introduced almost 20 years ago [26]. By removing, by study design, the intraventricular and infratentorial ICH, we considered that use of ICH score to stratify patients would be detrimental, and as such, we chose to record neurological admission status by GCS score.

While the GCS score was not primarily intended to be used in ICH, its role as a predictor of ICH outcome has been previously established [14,27]. We chose the GCS interval for inclusion in the study between 7 to 13 points, as in our institution, we do not consider suitable surgical candidates patients with ICH and GCS ≤ 6 or patients with good neurological status having GCS ≥ 14.

Our study is different from most research to date as it bases its allocation of treatment on an informed—yet subjective—decision by the next of kin. Due to this, we expected to observe some bias, especially towards the surgically treated arm. However, the number of cases was slightly higher in the medical group, and no significant differences between the two study groups were seen, which we believe validates our study.

The mean age of patients was 63 years, which was similar to that reported by other studies [28].

To date, no guidelines exist regarding surgical indications, the timing of surgery, or surgical techniques for the treatment of ICH [16]. The STICH I and STICH II trials revealed no clear benefit of surgery or early surgery over conservative treatment. Nevertheless, an ulterior analysis of surgical results showed that only 16% (STICH I) and 17% (STICH II) of patients were indeed operated on within 12 h from ictus [29].

Other authors have reported better results in patients operated on earlier than 12 h from ictus [30,31]. While still aiming to achieve rapid decompression, we chose to include all patients who met the inclusion criteria, regardless of the time passed from ictus. In this way, we allowed cases with a later onset of neurological status degradation, late-referral cases, and patients with difficult-to-contact relatives into this study.

Mortality was not significantly affected by older age in both the medically and surgically treated groups. While this outcome contradicts the findings of a meta-analysis by Gregson et al. [17], from which we adapted our surgical indications, similar results were reported in a recent study by Sondag et al. [22]. This seemingly confirming that older age should not be used as an exclusion criterion for surgically treating ICH patients [22].

Sondag et al. described no difference in mortality between minimally invasive surgically treated superficial and deep-seated ICH [22]. We seem to have found similar results, but we cannot conclude due to the limited number of patients.

In our surgical-indication group with ICH volumes of 30 to 70 mL, we found no correlation between mortality between moderate and large ICH volume. However, this result is most likely because of the relatively large ICH volumes used in this study and is similar to the results reported by Gregson et al. [17].

When comparing mortality between surgically and medically treated groups, we found that surgery is not inherently beneficial for all patients in our surgical study group. Still, despite including a limited number of cases, we observed that, in the subgroup of patients with moderate neurological impairment (GCS 10–13) and having moderate ICH volumes (30–50 mL), surgery lowered mortality when compared with medical treatment. While this post-hoc analysis is limited, we believe it shows that surgery still has a role, albeit limited, and reconfirms the similar results seen in the meta-analysis of Gregson et al. [17].

Nevertheless, our results are partially contradictory with those of the study performed by Safatli et al., which suggests an ICH volume of larger than 30 mL and a GCS score of fewer than 11 points to be significant predictors of mortality [24].

Our study shows that surgery, despite providing some good results for some ICH patients, still seems insufficient in lowering mortality even in the short term among overall ICH patients. Disrupting secondary brain injury using various neuroprotective techniques and compounds [16,32,33] and limiting surgical indications are likely critical components for more adequate ICH treatment strategies.

Even more, perhaps the classic medical and surgical treatment options have reached their limits. In the future, new areas of research, especially in molecular biology, will emerge that could ensure a critical leap forward in the management of this devastating pathology. In this sense, studies related to the role of extracellular vesicles as communication factors and intercellular transport for both normal and pathological tissues have been published. Exosomes, as circulating vesicles, have the role of transporting molecules specific to the cells of origin. These molecules are involved in cell regeneration, immune response, and new vascular supply by angiogenesis [34]. Exosomes from mesenchymal stem cells seem to play an essential role in the process of neuroregeneration, especially after hemorrhagic stroke [35,36].

We are aware that our study had a limited number of patients, with unavoidable selection bias, and study data are limited, especially regarding follow-up. Despite these limitations, we believe that our results showing better outcomes in moderately sized ICH and moderately neurologically impaired patients are encouraging and should prompt further research and better prospective and randomized studies on this subgroup of ICH patients.

## 5. Conclusions

Surgery does not appear to produce superior treatment outcomes for all forms of ICH. Nevertheless, some of our limited data still apparently confirms previous research indicating potential benefits of surgery in patients with GCS scores of 10 to 13 points and ICH volumes of 30 to 50 mL

## Figures and Tables

**Table 1:** Comorbidities in Group A (surgical) and B (medical).

Associated Comorbidities	Group A	Group B
High blood pressure	72.30% (*n* = 47)	69.01% (*n* = 49)
Cardiac disease *	33.84% (*n* = 22)	35.21% (*n* = 25
Diabetes	23.07% (*n* = 15)	26.76% (*n* = 19)
Obesity	21.53% (*n* = 14)	14.08% (*n* = 10)
Alcohol abuse	4.61% (*n* = 3)	7.42% (*n* = 5)

* cardiac disease includes heart failure, cardiac dysrhythmias, valvular heart disease.

**Table 2:** Mortality in the groups with surgical indications (Group A and B).

Morality	Group A (Surgical)	Group B (Medical)	
Total	45 (100%)	50 (100%)	
Superficial	30 (66.66%)	27 (54%)	*p* = 0.879
Deep	15 (33.34%)	23 (46%)	
30–50 mL	20 (44.45%)	27 (54%)	*p* = 0.4135
50–70 mL	25 (55.55%)	23 (46%)	
7–9	33 (73.33%)	24 (48%)	*p* = 0.0132
10–13	12 (26.67%)	26 (52%)	

**Table 3:** Relationship between mortality, admission Glasgow Coma Scale (GCS) and intracerebral hemorrhage (ICH) volume.

Mortality (Patients)	GCS 7–9/30–50 mL	GCS 7–9/50–70 ml	GCS 10–13/30–50 ml	GCS 10–13/50–70 ml
Group A (surgical)	14	19	6	6
Group B (medical)	13	11	14	12
*p*	1	0.157	0.0294	1

GCS—Glasgow coma score.

## Data Availability

Study data is available on request.
